# Sensitization of Carboplatinum- and Taxol-Resistant High-Grade Serous Ovarian Cancer Cells Carrying p53, BRCA1/2 Mutations by Emblica officinalis (Amla) via Multiple Targets

**DOI:** 10.7150/jca.36919

**Published:** 2020-01-29

**Authors:** Alok De, Archana De, Ramratan Sharma, William Suo, Mukut Sharma

**Affiliations:** Kansas City VA Medical Center and Midwest Veterans Biomedical Research Foundation, Kansas City, MO 64128, USA

**Keywords:** high-grade serous ovarian cancer, Amla, resistant, angiogenesis, metastasis, sensitize, mutation

## Abstract

**Background:** Ovarian cancer (OC), the most lethal gynecologic malignancy, is highly resistant to current treatment strategies. High-grade serous epithelial ovarian cancer (HGSOC) cells with increased somatic mutations and genomic instability and the resulting heterogeneous mutant phenotypes are highly resistant to therapy. Plant-derived natural products, including Amla (*Emblica officinalis*) extract (AE), have demonstrated potent anti-neoplastic properties. Recently we demonstrated that AE inhibits cell growth and the expression of angiogenic factors in OVCAR3 and SKOV3 OC cells *in vitro* as well as in xenografts *in vivo*. The goal of this study was to determine the anti-proliferative, anti-angiogenic and anti-metastatic effects of AE on carboplatinum- and taxol-resistant HGSOC cells carrying p53, BRCA1/2 mutations.

**Methods:** Anti-proliferative and anti-metastatic effects of AE on recently characterized carboplatinum- and taxol-resistant HGSOC cells (TOV3041G, OV866(2), OV4453 and, OV4485) was determined using the MTT, migration, invasion and spheroid assays i*n vitro.* To understand the mechanism of AE-induced changes in angiogenesis-related hypoxia-inducible factor 1α (HIF-1α) and insulin growth factor receptor 1 (IGF1R), and EMT-associated SNAIL1 and E-cadherin proteins were studied using immunostaining and Western blotting. *In vivo* effects of AE were determined using mouse xenograft tumor model of OC developed by subcutaneous injection of OV4485 cells that carry mutant p53 and BRCA1, most aggressive and resistant among HGSOC cell lines used in this study. Tumor growth was measured using morphometry. Immunostaining and Western blotting were used to determine changes in Ki67 (proliferation marker), CD31 (angiogenesis marker) as well as changes in HIF-1α, IGF1R, SNAIL1 and E-cadherin proteins.

**Results:** AE significantly attenuated migration and invasiveness properties of all tested HGSOC cell phenotypes (P≤0.001), significantly reduced the expression of HIF-1α, IGF1R, and SNAIL1 and increased the expression of E-cadherin in all tested HGSOC cell lines (P=<0.05). Oral administration of AE for 4 weeks caused a significant regression of mouse xenograft tumor (>60%) that derived from OV4855 cells and decreased the expression of endothelial cell antigen-CD31, HIF-1α, IGF1R and SNAIL1 and increased the expression of E-cadherin in tumor tissues.

**Conclusions:** AE sensitizes platinum- and taxol-resistant heterogenous HGSOC cells carrying mutations in p53, BRCA1/2 genes, and attenuates their malignant characteristics through targeting key signaling mechanisms of angiogenesis and metastasis. AE is a potential adjunct therapeutic agent for treating resistant, mutant, heterogenous OC.

## Background

Ovarian cancer (OC) is the fifth leading cause of cancer-related deaths and accounts for more deaths than any other cancer of the female reproductive system. OC often escapes early diagnosis and develops resistance to treatment resulting in poor prognosis that is closely associated with origins of the cancer 1. Resistant high-grade serous ovarian cancers (HGSOC) are aggressive and resistant to current therapies and are sustained by a microenvironment that promotes angiogenesis 2. Multiple genetic and epigenetic modifications as well as epithelial-to-mesenchymal transition (EMT) augment the survival, growth and metastasis of aggressive HGSOC 3. Specifically, angiogenesis is potentiated by hypoxia which is a common characteristic of many aggressive carcinomas 4. Hypoxia-inducible factor 1α (HIF-1α), a transcription factor, is a key mediator of tumorigenesis through activation of genes associated with angiogenesis and metastasis. Over-expression of HIF-1α is associated with invasive phenotypes and aggressive nature of OC cells and poor survival of patients with OC 5. Hypoxia induces HIF-1α and insulin-like growth factor 1 receptor (IGF1R) that independently or together induce growth, transformation, metastasis and angiogenesis 6. High rate of metastasis observed in OC begins with EMT, a critically significant process in invasive cancers 7. EMT activates a zinc finger protein transcription factor named SNAIL1 that promotes the invasive nature of human OC cells through downregulation of E-cadherin 8. These distinct but interconnected processes drive the growth and spread of resistant cancer. Simultaneous attenuation of these factors may be the key to minimize the aggressive nature of cancer cells.OC is characterized by a variety of mutations and heterogeneity that add to its resistant nature 9-13. Therefore, novel therapeutic approaches are needed to simultaneously target processes such as angiogenesis and EMT for an effective control of resistant cancer cells with diverse phenotypic characteristics. Adjunct agents that modulate multiple cellular processes may improve the efficacy of current therapeutic agents and overall clinical outcomes. Plant-derived products are known to have anti-tumor effects with low toxicity 14. A plant-derived compound has been shown to decrease mutation rate in tissues exposed to mutagen 15. In addition to its antioxidant effects, Amla (*Emblica officinalis*) (fruit, leaves, bark, root) extract (AE) has anti-neoplastic properties 14 and inhibits the proliferation of a variety of cancer cells *in vitro* including OC cells 14, prevents DNA damage induced by carcinogens and mutagens 14 and causes tumor regression in mouse xenograft model 14, 16, 17.

The objective of the present study was to determine whether AE can sensitize highly aggressive, mutant, metastatic and resistant heterogenous HGSOC cell lines (Table 1) with mutations in multiple genes 11. Our results show that treatment with AE attenuated proliferation, migration and invasiveness properties of all tested HGSOC cell phenotypes *in vitro* and caused >60% decrease in xenograft tumor size *in vivo*.

## Materials and Methods

### Ethical Statement

All animals were maintained according to standard guidelines of the American Association for the Accreditation of Laboratory Animal Care. The study was approved by the Institutional Animal Care and Use Committee of the Kansas City VA Medical Center (Kansas City, MO). Research described hereunder was conducted in agreement with ethical standards according to the Declaration of Helsinki, National and International guidelines.

### Cell culture and reagents

Dr. Mes-Messon, Montreal, Canada kindly gifted all HGSOC cell lines used in this study. As shown in Table 1 these HGSOC cell lines (i) are heterogenous, (ii) have a variety of different and important characteristics of the HGSOC disease in which p53 gene is non-functional - either mutated or silenced, (iii) do not harbor somatic mutations in KRAS, BRAF, ARIDIA, CTNNB1 or PIK3CA that have been previously shown to associate with low serous epithelial cells (29), and (iv) do not show high expression of HER2. These cells were recently characterized and, OV4485 are reportedly the most aggressive among these cell lines 11. Cells were maintained in ovarian surface epithelial Medium (OSEM, Wisent Bioproducts, Quebec, Canada) supplemented with 10% fetal bovine serum (ThermoFisher Scientific, Waltham, MA, USA) and penicillin-streptomycin (complete OSEM) at 37°C in 5% carbon dioxide and 7% oxygen. AE stock solution was prepared by dissolving AE tablets (Himalaya USA, Sugarland, TX) in endotoxin free sterile water (10 mg/mL) and filtering through a 0.22 µm cellulose acetate membrane 16, 17.

### Treatment

TOV3041G, OV866(2), OV4453 and OV4485 cells (9,000 cells/well in 100 µl in 96 well plate) or (50,000 cells/well in 1 ml of 24-well plates) in complete OSEM were treated with AE (0-800 µg/ml; each in triplicates) for 24-96 hours at 37°C.

### Cell proliferation

Cell proliferation was assessed using [3-(4,5-dimethylthiazol-2-yl)-2,5-diphenyltetrazolium bromide)] (MTT) assay. Control and treated cells were incubated with MTT (0.1 mg/well, Millipore-Sigma) for 4h at 37°C. The formazan crystals formed were solubilized in isopropanol (100 µl) and optical density was measured at 560 nM. The number of functionally active cells was calculated from optical density values for untreated and treated groups. Results are presented as ± standard error means (±SEM) of six experiments performed in duplicates for each treatment condition.

### Invasion assay

Cells (7×10^4^/well) suspended in serum-free OSEM (250 µl) were layered on 24- Transwell (Corning®, NY, USA) permeable support membranes pre-coated with Matrigel matrix (100 µl/well, Corning®, NY, USA). Complete OSEM alone or complete OSEM (600 µl/well) containing AE (400-500 µg/ml) was added to wells. Preliminary work showed that OV866(2) moved faster and OV4485 cells moved slower than other cells. Therefore, OV866(2) cells were fixed at 6h and OV4485 cells at 24h in cold 100% methanol, stained with 0.1% crystal violet and photographed. For quantitative analysis of cell migration, five randomly chosen different areas of crystal violet-stained cells were counted. Results are presented as the number of cells ± SEM of three experiments performed in duplicates for each treatment condition.

### Spheroid assay

Previously described spheroid assay was used to determine the effect of AE on the ability of HGSOC cells to loosen aggregates of spheroid structures 11. Briefly, 4,000 cells were suspended in 16 µl of complete OSEM with or without AE (400 µg/ml for all cell lines and 500 µg/ml for OV4485 cell line) and placed on the lid of tissue culture petri dishes. Spheroid formation was assessed after 7 days of incubation in 7% O_2_, 5% CO_2_ at 37°C. This experiment was repeated four times.

### Migration assay

Scratch wounds were made to 80-90% confluent TOV3041G, OV866(2), OV4453 or OV4485 cells, treated with AE (0-800 µg/ml) and scratched areas were photographed at 0, 6, 24 and 48h using EVOS XL Core Cell Imaging System (ThermoFisher Scientific). Since OV4485 cells migrate slowly,these cells were treated up to 72h and photographed at 0, 24, 48 and 72h. The relative migration gap distance was calculated using the equation: the relative migration distance (%) = 100 (A-B)/A, where A and B are the widths of the scratch area before and after incubation, respectively. Results are shown as mean relative migration gap ± SEM. from six independent experiments.

### Immuno-staining

HGSOC cells and xenograft tumors were fixed in 4% buffered formaldehyde (pH 7.4). Cells and tissue sections were incubated with 1:100 diluted antibodies to HIF-1α, SNAIL1, E-cadherin (Cell Signaling Technology, Boston, MA, USA) or IGF1R (EMD Millipore, Billerica, MA, USA) overnight at 4°C. Immuno-staining was performed using ImmPRESS HRP reagent kit (Vector Laboratories, Burlingame, CA, USA). Cells and tissue sections were counterstained with hematoxylin. A Leica digital microscope was used for imaging to determine the percent immuno-stained cells (immuno-stained cells/total number of cells x 100). Two investigators, blinded to sample-source, independently counted immuno-stained cells. Cells and tissue sections were first examined at 5x magnification to identify immuno-positive regions in cultured cells and tumor sections. Five different areas of cultured cells and tumor sections were selected at random and evaluated microscopically at a 40x objective magnification. Immuno-stained cells and total cells were counted and the percentage of cells with immunolabeling was calculated. Cultured cells from four independent experiments were used. For tissue immuno-staining five mice were used in each group.

### Western blotting

Ten microgram total protein was used for SDS-PAGE followed by electro-transfer to a nitrocellulose membrane. The membrane was incubated overnight at 4°C with 1:1000 diluted primary antibodies to HIF-1α, SNAIL1, E-cadherin or IGF1R. Anti β-actin 1:10000 dilution (Abcam) was used as loading control. The membrane was then incubated with 1:10000 diluted HRP-conjugated secondary antibodies (Millipore-Sigma) at room temperature for 1h. Immunoreactivity was detected using enhanced chemiluminescence reagent (GE Healthcare Bio-Sciences, Marlborough, MA). The results are presented as mean ± SEM of three experiments. β-actin was used as the loading control.

### Xenograft tumor studies

Nude mice (nu/nu genotype, Harlan Laboratories, Madison, WI) were maintained at the AAALAC accredited Animal Care Facility of KC VAMC. Eight-week-old nude mice were maintained on water and food *ad libitum* in a pathogen free environment with a 12 h light and 12 h dark cycle. In previous *in vivo* studies we used OVCAR3 and SKOV3 cells. These cells are now considered less aggressive. OVCAR3 cells express the R248Q mutant p53 and SKOV3 cells do not express p53 protein or mRNA (29). In the present studies we intended to determine whether AE has any effect on xenograft tumor derived from resistant/aggressive cells with mutation(s) in different/additional genes (e.g. BRCA). Therefore, we used OV4485 cells which is a BRCA1 mutated cell line resistance to carboplatinum (Table 1). Fleury et al showed rapid growth of tumors derived from OV4485 cells and poor survival of mice compared to tumors developed from BRCA2 mutated OV4453 cells 13. OV4485 cells (5x10^6^) with Matrigel (1:1 ratio volume) were injected subcutaneously into the right rear flanks of eight weeks old female mice. Mice were monitored every day for tumor mass that was visible at 2 months after cell injection. Once the tumor mass was visible, mice were randomly divided into two groups: control and treated. Treatment group received AE (100 mg/kg body weight/day in 10% sucrose) during night time only as described previously 16, 17. Control group received 10% sucrose solution only during night time. Mice were weighed twice/week. Tumor size was measured with digital caliper once every week and volume calculated using the formula: tumor volume = length x width x 0.5 width. After 4 weeks of AE treatment mice were euthanized. Tumors were removed and processed for analysis (immunohistochemistry and Western blotting). Five mice in each group (control and treated) were used based on our previous experience 11, 12. No adverse effect was noted due to AE treatment.

### Statistical analysis

All experiments were conducted for three replicates. All data are expressed as the mean ± standard error means (SEM). Significance was tested using unpaired, two-tailed Student's t-Test with unequal variance and ANOVA with Bonferroni post hoc test. P≤ 0.05 was considered significant.

## Results

### AE attenuated the proliferation of HGSOC cells in a dose- and time-dependent manner

TOV3041G (non-mutated, resistant), OV866(2) (TP53 mutation, resistant), OV4453 (TP53, BRCA2, CSMD3 and RB1 mutations, resistant) and OV4485 (TP53 and BRCA1 mutations, resistant) were cultured as outlined under Methods and were treated with AE (100-800 µg/ml) for 24-96 hours to assess cell proliferation and viability using the MTT assay.

**Figure 1:**
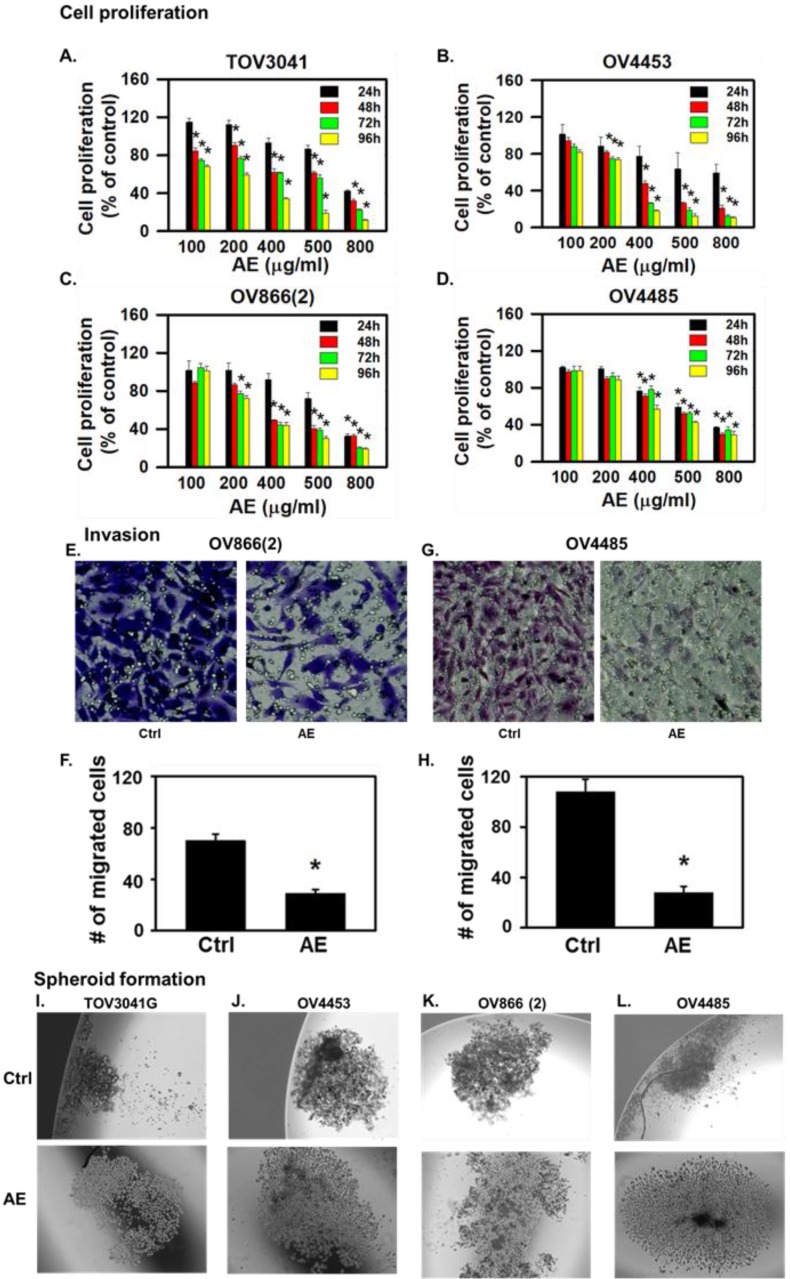
** AE treatment inhibited cell proliferation, invasiveness and spheroid formation in highly aggressive, resistant, metastatic mutant high-grade serous ovarian cancer cells. (A-D)** Time- and dose- dependent effects of AE on proliferation of **(A)** TOV3041, **(B)** OV4453, **(C)** OV866(2) and **(D)** OV4485 cells. Cells were treated with different doses of AE for 24-96 hours as shown and proliferation was assesses using the MTT assay. Results are presented as percent of untreated control cells (mean ± S.E.M, n=6 independent observations, *, P<0.05). **(E-H)** AE inhibited invasiveness of OV866(2) and OV4485 cells. **(E-F)** AE (400 µg/ml) inhibited the invasiveness of OV866(2) after incubation for 6 h and **(G-H)** AE (500 µg/ml) inhibited the invasiveness of OV4485 cells for 24 h determined using the Transwell assay. Representative images (20x magnification) show OV866(2) and OV4485 cells that crossed to the basal side of the membrane indicating invasiveness. **(F, H)** Number of migrated cells from randomly chosen five areas of crystal violet-stained cells (mean ± SEM of 3 independent experiments each in duplicate. *, P≤0.05 compared with control). **(I-L)** AE decreased the formation of compact spheroid formation by TOV3041G, OV866(2), OV4453 and OV4485 cells. Representative images show loosened spheroids after AE treatment. C=control, AE=Amla extract.

AE inhibited the proliferation of all cell lines in a dose- and time-dependent manner. At 24 hours, lower concentrations of AE (100 µg/ml and 200 µg/ml) caused 20-30% inhibition of cell proliferation of TOV3041G, OV866(2) and OV4453 (**Fig. 1A-C**) (but not OV4485 cells) compared to untreated control cells (**Fig. 1D**). Half maximal dose for three cell lines at 48 h was 400 µg/ml except OV4485 that required 500 µg/ml for half maximal effect (**Fig. 1A-D**). Higher doses of AE (500-800 µg/ml) caused significant inhibition at 24-96 h. These results suggest that AE has anti-proliferative effect on resistant, mutated, heterogenous HGSOC cells and that BRCA1 mutated OV4485 cells (isolated after carboplatin/taxol treatment, Table 1) required higher dose of AE and for longer duration.

### AE inhibited malignant properties (invasiveness, spheroid formation and migration) of HGSOC cells *in vitro*

Invasive/aggressive cells constitute one of the characteristics of cancer metastasis 18. Therefore, we compared the effect of AE on invasiveness of OV866(2) and OV4485 cells by the invasion assay using Transwells. AE treatment attenuated the invasiveness of OV866(2) cells (P=0.02) at 6 hours and OV4485 cells (P=0.01) at 24 hours (**Fig. 1 E-H**) highlighting the potent effect of AE on cells with differences in mutations and invasiveness.

Spheroid formation represents one of the major three-dimensional (3D) *in vitro* models for cancer studies that serve as an intermediate approach between *in vitro* cancer cell line cultures and *in vivo* tumors 19. Therefore, we studied spheroid formed by TOV3041G, OV866(2), OV4453 and OV4485 cells and found that spheroid formed by these cells were compact (**Fig. 1I-L**). AE treatment loosened the spheroids formed by each of the tested HGSOC cell line (**Fig. 1I-L**). These results suggest that AE inhibits the formation of compact spheroid by HGSOC cells.

**Figure 2:**
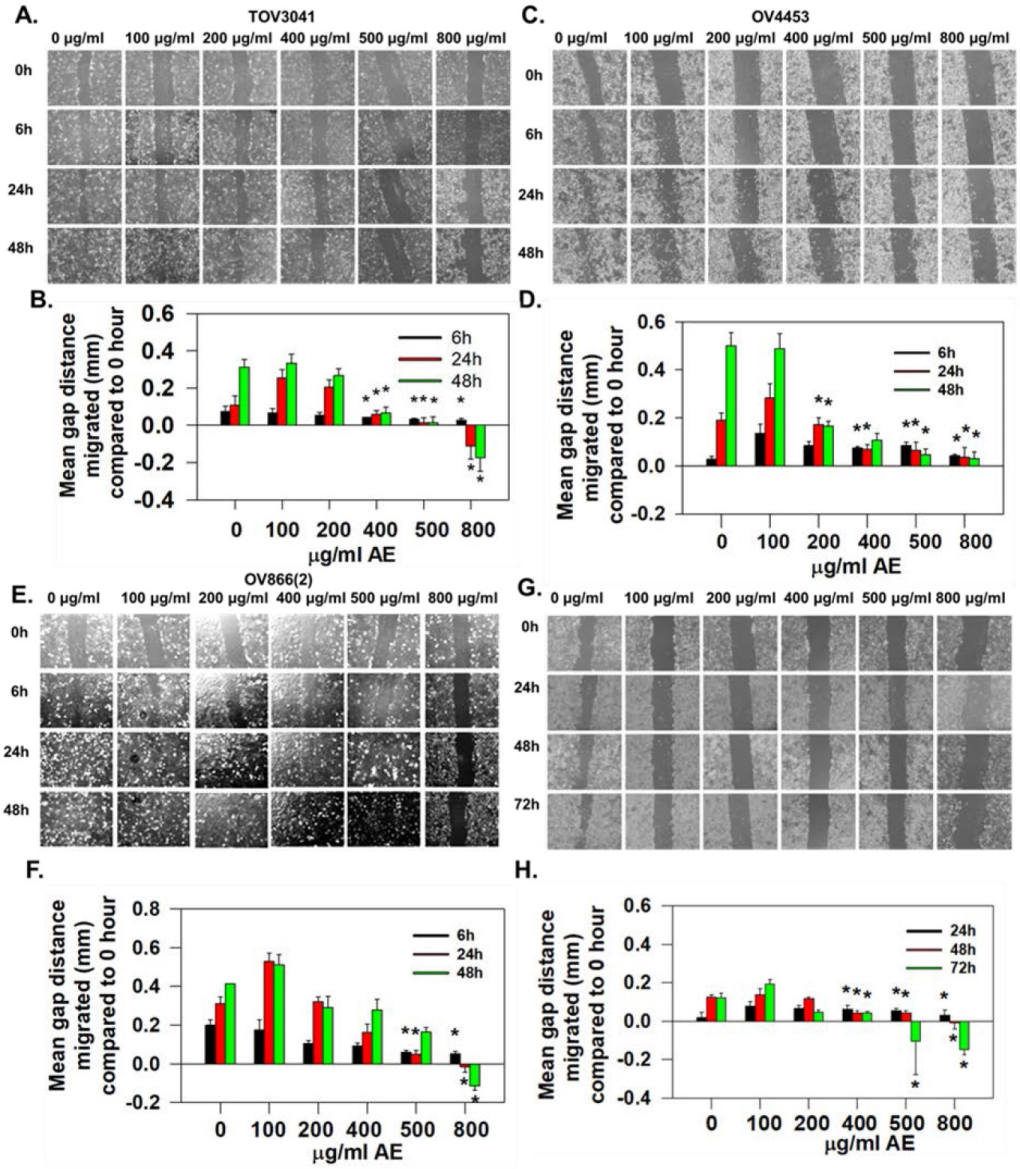
** AE inhibited wound-healing by HGSOC cells in a time- and dose-dependent manner. (A-H)** TOV3041, OV4453, OV866(2) and OV4485 cells were scratch-wounded at 90% confluency and treated with 100-800 µg/ml AE and further incubated for 48-72 h. Gap distances were measured, normalized using an untreated control. **(A, C, E)** Representative images showing the inhibitory effect of AE on wound-healing migration of **(A)** TOV3041, **(C)** OV4453, and **(E)** OV866(2) cells captured at 0, 6, 24, and 48 h. **(G)** Representative images showing the inhibitory effect of AE on wound-healing migration of OV4485 cells captured at 0, 24, 48 and 72 h.** B, D, F** and **H** Quantitative analysis of the results presented under **(A, C, E, G)**. Results are shown as mean + S.E.M. from 6 independent experiments. *, P≤0.05 compared with 0 hour was considered significant.

Cell migration is another major characteristic of metastasis 18. Therefore, we studied the effect of AE on migration of TOV3041G, OV866(2), OV4453 and OV4485 cells using the scratch wound healing assay. Our results show that AE at lower concentration partially inhibited migration (**Fig. 2A, C, E, G**) and this effect of AE on migration of TOV3041G, OV866 (2), OV4453 and OV4485 cells varied in dose- and time-dependent manner. A comparison of relative gap distances after AE treatment is shown in **Fig. 2B, D, F, H**).

Migration of TOV3041 cells was not affected by any concentration of AE for 6 h but only 800 µg/ml AE significantly inhibited migration after 24 h of incubation (P=0.003). AE at 400 µg/ml, 500 µg/ml and 800 µg/ml significantly (P=0.013, P=0.002 and P≤0.001, respectively) inhibited migration at 48 h incubation (**Fig. 2A and B**).

Migration of OV4453 cells remained unaffected by all concentrations of AE at 6 h and 24 h. AE at 200-800 µg/ml caused significant inhibition (P≤0.001) of migration at 48 h incubation (**Fig. 2C** and **D**).

Migration of OV866(2) was affected only at AE concentrations of 500 µg/ml (P=0.02) and 800 µg/ml (P=0.002) by 6 h. AE at 800 µg/ml significantly (P≤0.001) blocked the migration at 24 h and 48 h (**Fig. 2E** and **F**).

OV4485 cells appeared to be slow moving and migration was unaffected by all tested concentration of AE up to 24 h of incubation. At 48h, 400 µg/ml (P=<0.02), 500 µg/ml and 800 µg/ml (P=<0.001) of AE significantly inhibited migration. At 72 h, 400 µg/ml (P=<0.02), 500 µg/ml and 800 µg/ml (P=<0.001) of AE inhibited migration significantly (**Fig. 2G** and **H**).

OV866(2) cell line has been shown to have the highest anchorage-independent growth and cell invasion compared to the TOV3041G, OV4453 and OV4485 cell lines (13). Present results also show OV866(2) cells to have faster invasion rate compared to OV4485 cells and AE reduced invasion rate in both cell lines. These results suggest potential beneficial effect of AE for reducing tumor peritoneal metastasis and invasion. Malignant properties of all cell lines were attenuated by AE however; longer treatment with higher dose were required for more resistance cells (OV4485).

### AE down regulated the expression of HIF-1α in HGSOC cells

HIF-1α plays a significant role in invasion and metastasis of cancer cells and AE treatment attenuated invasion and metastatic properties of HGSOC cells, therefore, we determined the effect of AE on the expression of HIF-1α protein in all HGSOC cell lines.

**Figure 3:**
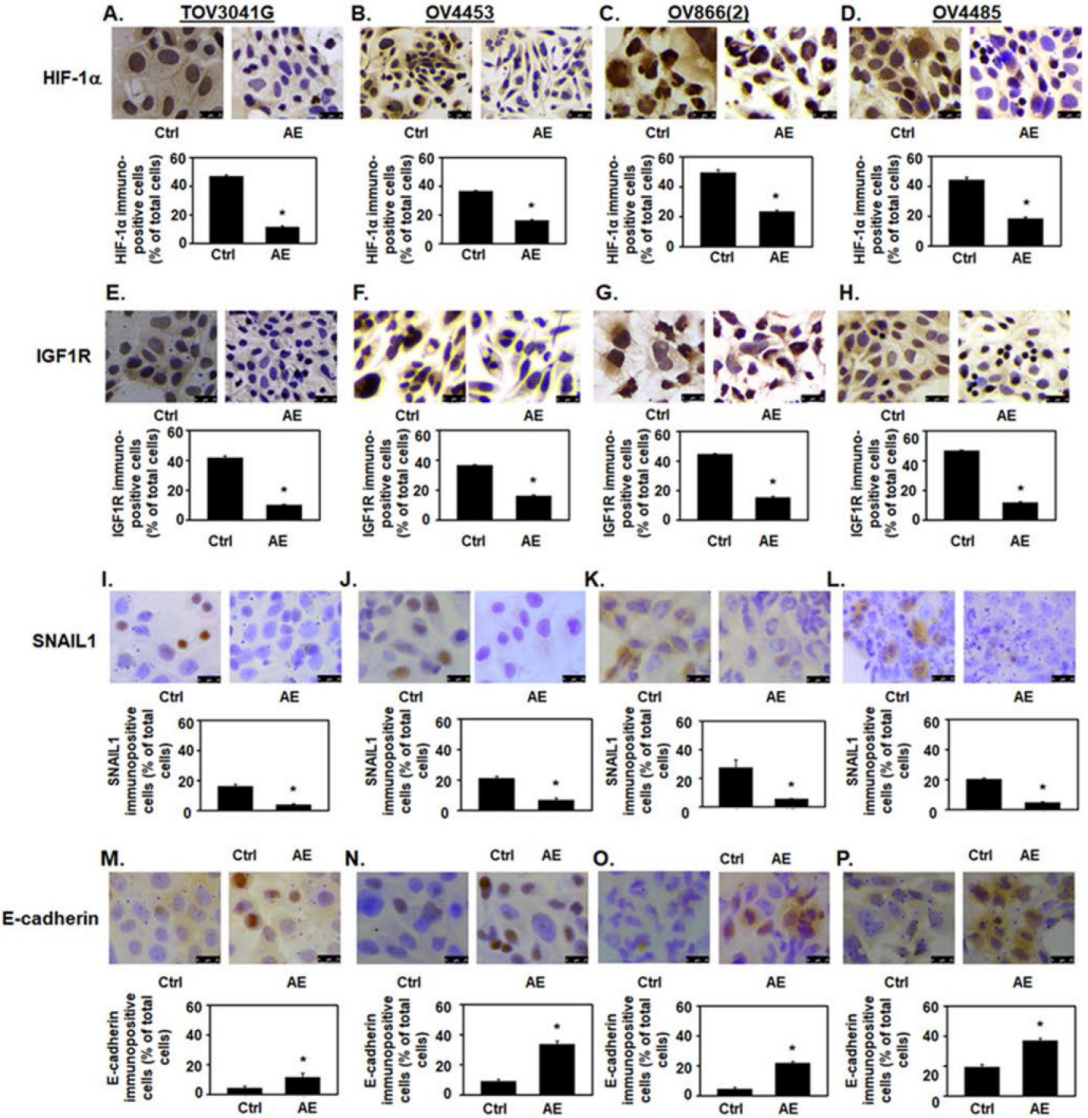
** AE downregulates the expression of HIF-1α, IGF1R, and SNAIL1, and upregulates the expression of E-cadherin in HGSOC cells - (A, E, I, M) TOV3041G, (B, F, J, N) OV4453, (C, G, K, O) OV866(2) and (D, H, L, P) OV4485**. Confluent cells were incubated with AE (400 µg/ml for TOV3041G, OV4453, OV866(2) and 500 µg/ml OV4485) for 48 hours and analyzed using immunocytochemistry. Representative photomicrographs of 4 experiments are shown. Number immuno-stained cells were counted and calculated as percent of total cells. Results show decreased immuno-staining for HIF-1α protein **(A-D)**,** (E-H)** IGF1R protein and **(I-L)** SNAIL protein. In contrast immuno-staining for E-cadherin protein was increased **(M-P)**. Results are presented as bar graphs showing Mean ± SEM, *, P≤0.05 compared with control group. Bar=25 µm. Ctrl=control, AE=Amla extract.

**Figure 4:**
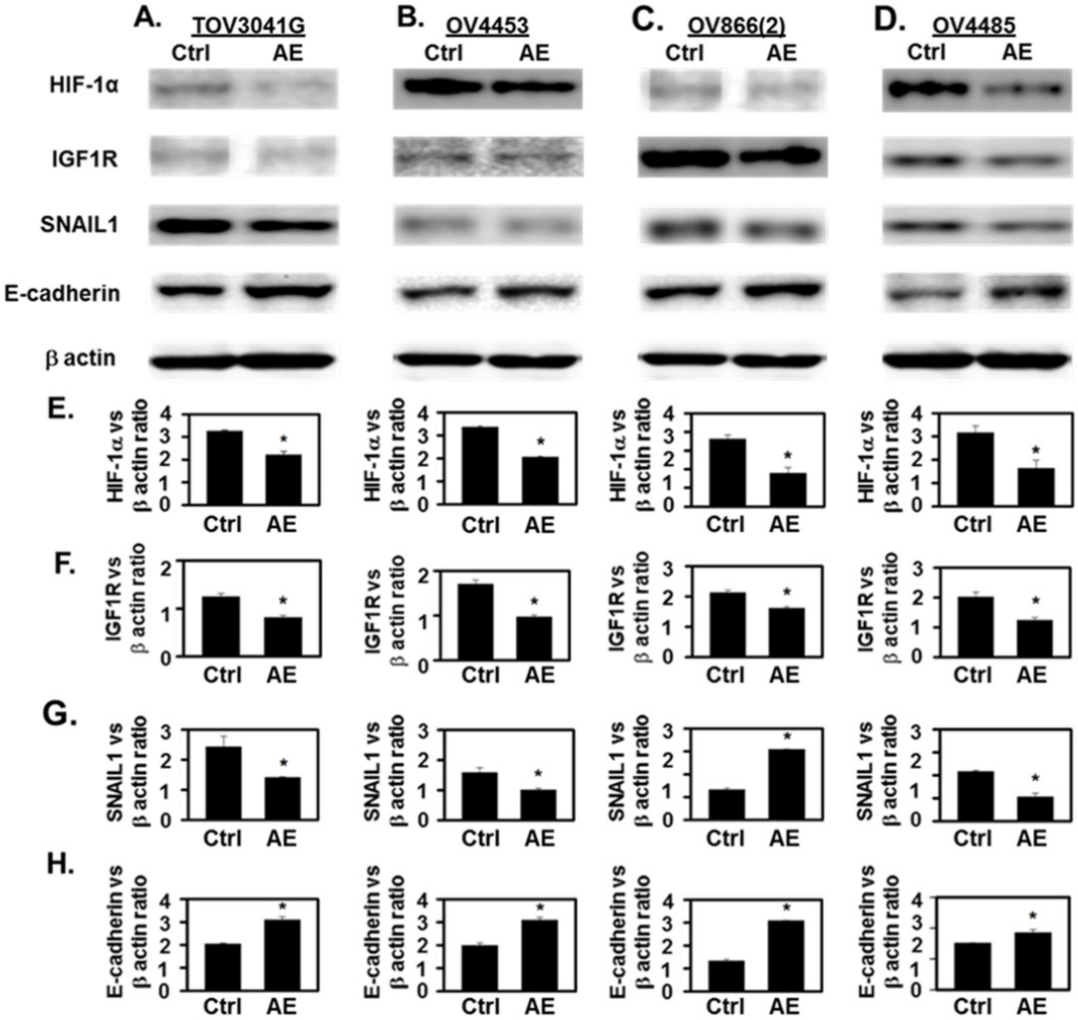
** Incubation with AE downregulated the expression of HIF-1α, IGF1R and SNAIL1 and increased the expression of E-cadherin in HGSOC cells- TOV3041G, OV4453,** OV866(2) and OV4485. Confluent cells were incubated with AE for 48 hours and analyzed using SDS-PAGE and Western blotting as described. **(A-D)** Representative images in upper panels show that treatment with AE resulted in decreased expression of HIF-1α, IGF1R and SNAIL1 proteins. In contrast, AE-treated cells showed increased expression of E-cadherin protein. **(E-H)** Densitometry ratios of HIF-1α/β-actin, IGF1R/β-actin, SNAIL1/β-actin and E-cadherin/β-actin. Results are presented as Mean ± SEM of 3 experiments. *, P<0.05 vs. control. β-actin was used as the loading control. Ctrl=control, AE=Amla extract.

AE treatment decreased HIF-1α immunostaining intensity and the number of immuno-positive cells (P≤001) (**Fig. 3A-D**). In addition, AE treatment in HGSOC cells significantly reduced HIF-1α protein levels as measured by Western blot analysis (**Fig. 4A-D**, HIF-1α panel and **Fig. 4E**). Our results show that expression of HIF-1α was significantly reduced in all HGSOC cells studied, however, OV4485 cells required higher dose of AE (500 µg/ml) for significant reduction in HIF-1α expression.

### AE down regulated the expression of IGF1R in HGSOC cells

IGF-1R is expressed at high levels on the surface of several types of cancer cells and its activation by IGF and/or hypoxia has been shown to cause cells to grow and divide 20 21. We studied the effect of AE on the expression of IGF1R protein in HGSOC cell lines. Both immunocytochemistry and Western blot results showed significant decrease in the expression of IGF-1R protein in all tested HGSOC cells. AE treatment decreased the intensity of IGF1R immuno-staining and the number of immuno-positive cells (P=0.001, **Fig. 3E-H**). Western blot analysis also revealed significant inhibition of IGF-1R protein following AE treatment in TOV3041G (P=0.005), OV866(2) (P=0.002), OV4453 (P=0.007) and OV4485 (P=0.01) cells (**Fig. 4A-D**, IGF1R panel and **Fig. 4F**). These results suggest that AE down regulates angiogenesis in HGSOC cells as both HIF-1α and IGF1R are known to promote angiogenesis 4, 22.

### AE decreased the expression of metastasis-associated transcription factor SNAIL1 in HGSOC cells

Hypoxia upregulates SNAIL1 in OC cells 23. Therefore, we studied effect of AE treatment on SNAIL1 expression in HGSOC cells. Our results show a significant decrease (P≤0.001) in SNAIL1 protein expression in AE-treated HGSOC cells by immunostaining (P≤0.001) (**Fig. 3I-L**) and a significant decrease in SNAIL1 protein levels in TOV3041G (P=0.04), OV866(2) (P=0.03), OV4453 (P=0.01) and OV4485 (P=0.03) by Western blot analysis following AE treatment (**Fig. 4A-D**, SNAIL1 panel and **Fig. 4G**).

### AE upregulated the expression of E-cadherin in HGSOC cells

Hypoxia attenuates E-cadherin expression *via* up-regulation of SNAIL in OC cells 24 where SNAIL1 is a direct repressor of E-cadherin, therefore, we studied the effect of AE treatment on E-cadherin protein expression in HGSOC cells. Our results show significant upregulation of E-cadherin expression (P=0.03 to P<0.001) by immunostaining after AE treatment and an increase in E-cadherin protein levels by Western blot analysis in TOV3041G (P=0.002), OV866(2) (P=0.01), OV4453 (P=0.002) and OV4485 (P=0.02) cells (**Fig. 3M-3P** and **Fig. 4A-D**, E-cadherin panel and **Fig. 4H**). These results document that AE treatment modulates malignant properties of HGSOC cells through inhibition of SNAIL1 and activation of E-cadherin. Though Fleury *et al* did not detect E-cadherin in OV866(2) and OV4453 cells (13), present studies detected upregulation of E-cadherin following treatment with AE. Decreased expression of HIF-1α, IGF1R and SNAIL1 and increased expression of E-cadherin in these cells appeared to depend on the dose of AE. BRCA1 mutated cells required higher amounts of AE compared to other cells.

### AE inhibited tumor growth, HIF-1α, IGF1R, and SNAIL1 expression, but stimulated E-cadherin expression in HGSOC cell xenograft tumors *in vivo*

We used mouse xenograft model to study *in vivo* effects of AE treatment. After 60 days of subcutaneous injection of OV4485 cells in nude mice, when tumors grew to form visible masses, animals were divided into untreated control group, and AE treated group (N=5 mice/group). Mice in the untreated control group were fed 10% sucrose solution, whereas the treated group received AE in 10% sucrose solution for 28 days. Mice were sacrificed at 88 days after inoculation and tumors were excised.

**Figure 5:**
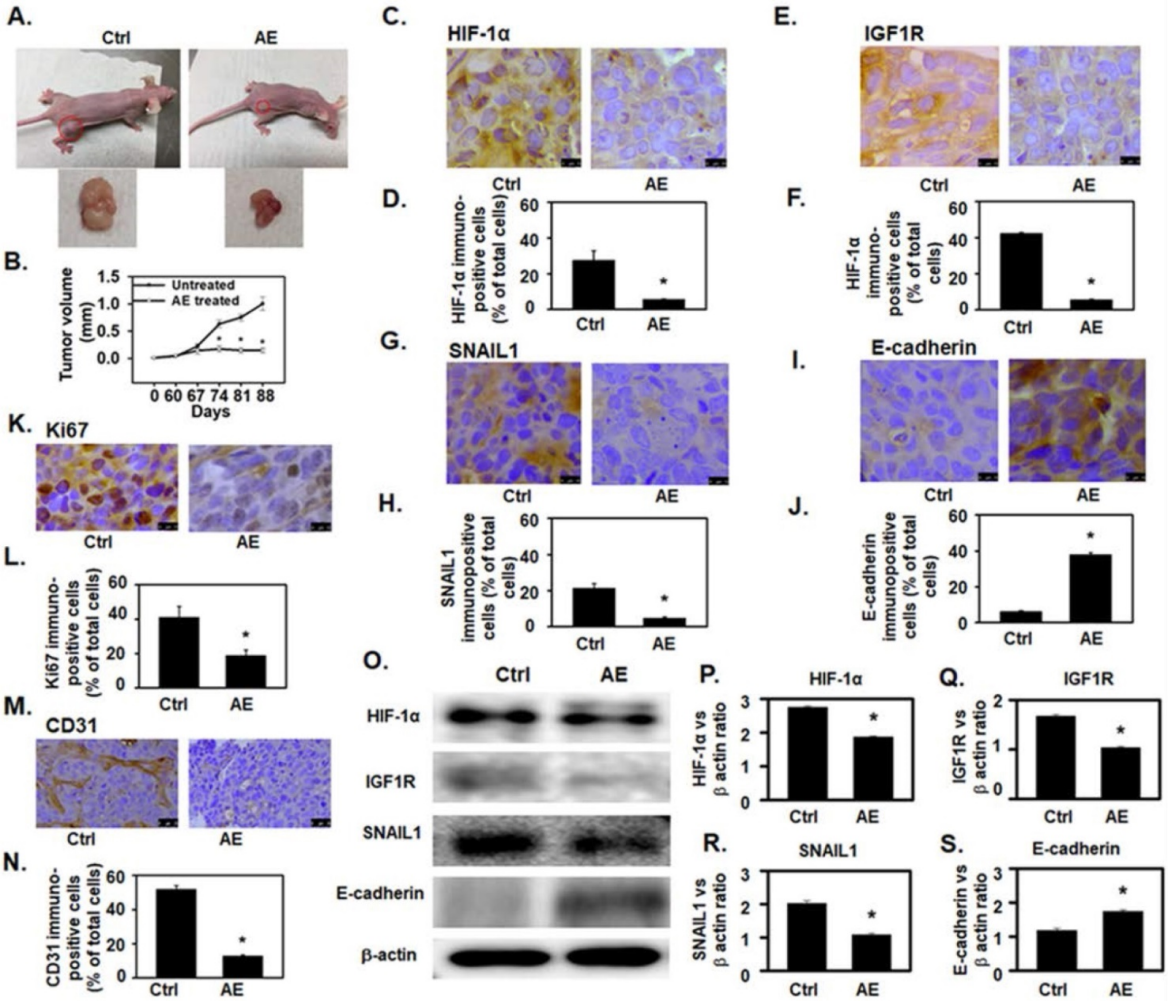
** AE attenuated OV4485 xenograft tumor growth in athymic nude mice and downregulated HIF-1α, IGF1R and SNAIL1 protein expression but upregulated E-cadherin protein expression. (A) Left upper:** Untreated Nude mouse bearing tumors, **left lower**: tumor from untreated mouse (control), **Right upper:** AE treated mouse. **Right lower:** tumor from AE-treated mouse. **(B)** Line graph showing change in tumor volume with time. Black line: untreated (control) tumor, Gray line: AE treated. **(C-N)** Immuno-histochemical analysis of xenograft tumor in untreated control and AE-treated mice. Results presented as bar graphs show immuno-positive cells as percent of total cell. **(C-D)** HIF-1α expression was decreased by AE treatment. Bar=10 µm. **(E-F)** IGF1R expression was decreased after AE treatment. Bar=10 µm. **(G-H)** Decreased expression of SNAIL1 in xenograft tumors after AE treatment. Bar=10 µm. **(I-J)** increased immuno-staining for E-cadherin in xenograft tumors after treatment with AE. Bar=10 µm. **(K-L)** Decreased immuno-histochemical expression of Ki67 positive cells in mouse tumor xenograft after AE treatment. Bar=10 µm. **(M-N)** AE treatment decreased the immuno-histochemical expression of CD31 positive cells in mouse xenograft tumors Bar=25 µm. **(O)** Representative Western blots showing a decreased expression of HIF-1α, IGF1R, SNAIL1 and increased expression of E-cadherin. β-actin was used as the loading control. (P-S) Western blotting results presented as densitometric ratios of, **(P)** HIF-1α/β-actin, **(Q)** IGF1R/β-actin, **(R)** SNAIL1/β-actin and **(S)** E-cadherin/β-actin. All results were obtained using tumor tissues from 5 mice in each group. Values are Mean ± SEM, *, P≤0.05 compared with control group. Ctrl=control, AE=Amla extract.

As compared to untreated control group, AE treatment significantly decreased (P=0.02) tumor size (**Fig. 5A-B**) without obvious adverse effects. To confirm our *in vitro* findings, xenografts from untreated control and AE treated mice were examined for HIF-1α, IGF1R, SNAIL1 and E-cadherin protein expression. The number of HIF-1α (P=0.006), IGF1R (P<0.001) and SNAIL1 (P=0.004) immuno-positive cells as percent of total number of cells was significantly reduced in AE treated group (**Fig. 5C-H**). The number of E-cadherin immunopositive cells was significantly increased (P≤0.001) in AE-treated group (**Fig. 5I-5J**).

Analysis of HIF-1α, IGF1R and SNAIL1 protein levels were decreased in AE-treated xenografts (**Fig. 5-O** panels HIF-1α, IGF1R, and SNAIL1 and **Fig. 5P-R** whereas intensity of E-cadherin protein band (P≤0.01) was significantly increased in AE-treated xenografts (**Fig. 5O** panel E-cadherin and **5S**).

AE treatment significantly decreased (P=0.01) the number of Ki67 (a cellular marker for proliferation) 24 positive cells (**Fig. 5K-L**) suggesting that cell proliferation was inhibited in xenograft tumors of AE treatment compared to untreated control.

In addition, angiogenesis in xenograft tumor was studied by measuring tumor vascularization in tissue sections stained for CD31 - an endothelial marker. CD31 expression was significantly reduced in AE treated xenograft compared to controls (**Fig. 5M**). In addition, microvessel density was also significantly reduced (P=0.004) in xenografts tumors from AE treated mice (**Fig. 5N**) as compared to untreated control.

## Discussion

Results outlined above show that AE sensitized highly aggressive, resistant, metastatic HGSOC cells with or without mutations in p53, BRCA1/2 genes. AE treatment inhibited cell proliferation, migration and invasion properties of HGSOC cells. AE treatment also reduced the size of the xenograft tumor in mice developed from the most aggressive of the cell lines (OV4485) used. Our results show for the first time that AE treatment simultaneously inhibited cell growth and modulated key molecules of metastatic pathways (HIF-1α, IGF1R, SNAIL1 and IGF1R and E-cadherin) in resistant mutant HGSOC cells.

Key features of aggressive carcinomas including uncontrolled cell proliferation, migration, invasion and metastasis, are characterized by gene instability and impaired cellular signaling 25. Spontaneous mutations in cancer cells can create phenotypically diverse populations of cells with varying mutations. Mutations including TP53, BRCA1, BRCA2, CSMD3 and RB1 are known to contribute to cancer cell migration, invasion and metastasis 26, 27. The malignant and resistant nature of OC depends on phenotypically distinct sub-populations of cells with genomic instability and diverse mutations 28. Previously we reported that AE treatment inhibits proliferation in OVCAR3 and SKOV3 cells. OVCAR3 cells express the R248Q mutant p53 and the SKOV3 is also a p53-mutant cell line which does not express p53 protein or mRNA 16, 17. However, these cells are now considered to develop non-aggressive OC with diverse mutations 29. Effect of AE treatment on heterogenous HGSOC is not known. Therefore, we selected four cell lines that have been characterized to reflect the heterogeneity of HGSOC. These included TOV3041G with no deleterious mutation, OV866(2) with TP53 mutation, OV4453 with TP53, BRCA2, CSMD3 and RB1 mutation, and OV4485 with TP53 and BRCA1 mutation. These cell lines were derived from samples collected at diagnosis or at the time of relapse, from either solid tissue (TOV) or ascites (OV) as described in Table ​1. The OV4453 cell line was derived from ascites from patients who had not received chemotherapy prior to surgery; whereas the OV866(2), TOV3041G and OV4485 cell lines were derived from samples obtained from recurring disease and thus the patients had been treated with chemotherapy before collection. OV4485 was reported to be the most aggressive among these cell lines. Results show that AE effectively inhibited growth, migration and invasiveness of heterogenous HGSOC cells. To further investigate the effect of AE on these HGSOC cells, we selected key molecules related to hypoxia, angiogenesis and EMT namely, HIF-1α, IGF1R, SNAIL1 and E-cadherin.

HIF-1α is involved in tumor cell proliferation, angiogenesis, metastasis, and chemotherapy resistance 6. Overexpression of HIF-1α is observed in cells expressing wild type p53 and is associated with undifferentiated high-grade tumors that are refractory to treatment resulting in higher mortality rate. Overexpression of HIF-1α under normoxia increases p53 activity and, HIF-1α is essential for developing p53 mutant mouse model 30 31. p53 mutations accelerate cancer cell growth, poor differentiation, poor prognosis and resistance to treatment 32. Both p53 and HIF-1 are mediators of cell adaptation to many stresses and are known to be involved in processes such as apoptosis, cell cycle control, and metabolism 33. AE treatment attenuates oxidative damage-induced mutation and downregulates HIF-1α expression in OC cells 34 16.

HIF-1α is overexpressed in BRCA1 and BRCA2 mutation carriers 35. BRCA1 interacts with HIF-1α and regulates HIF-1α stability 36. BRCA1 protein along with genetic and epigenetic changes of the BRCA1 gene is involved in the development of OC 37. Our results show that AE treatment reduces HIF-1α expression in all tested HGSOC cell lines.

HIF-1α, p53 and BRCA1 closely interact with IGF1/ IGF1R signaling 21. IGF1R expressed in malignant tumors plays a critical role in cell survival, invasion, metastasis, and angiogenesis 21. IGF1R activation by IGF-1/IGF-2 induces HIF-1α and, both IGF1R and HIF-1α may simultaneously inhibit cell migration and invasion 38 39. IGF1R interacts with wild type p53 21. IGF1R levels are elevated in BRCA1-inactivated OC cells and BRCA1 knockdown activates IGF1R expression in non-BRCA1-mutated OC cells 40. Results of the present study document that AE treatment downregulates IGF1R expression in all tested HGSOC cell lines.

IGF1/IGF1R signaling and HIF-1α regulate angiogenesis 21. Results of the present study demonstrate that AE treatment suppresses expression of angiogenesis-related proteins namely, HIF-1α and IGF1R in HGSOC cells and, also in HGSOC derived xenograft tumor. This anti-angiogenic effect of AE treatment on HGSOC-derived xenograft tumor is further supported by results showing downregulation of angiogenesis marker CD31.

EMT is an early cellular process in cancer metastasis initiated by gene mutations. Mutation in p53 increases SNAIL1 protein expression and activity; and promotes EMT in many cancers 41. SNAIL1 represses adhesion molecule E-cadherin and BRCA1 42 43. SNAIL1 expression is high in low BRCA1 triple negative breast cancer 44. Presently, AE inhibited SNAIL1 expression and upregulated E-cadherin expression in resistant heterogenous HGSOC cells. We have previously reported similar effects of AE causing inhibition of growth and proliferation and angiogenesis and metastatic properties of OC in OVCAR3 and SKOV3 cells 29.

Currently OC are treated using a combination of approaches including chemotherapy agents, surgery and radiation. Ideas for treating cancer using multiple agents to target multiple molecules/pathways, for overcoming resistance and turning cancer into a manageable disease are being considered. Several plant-derived products provide an economical way to target multiple cancer molecules/pathways simultaneously 45, 46 to complement and enhance effectiveness of currently available treatment regimens. Flavonoids and alkaloids in plant extracts have been used to overcome drug resistance 47. AE is known for antioxidant properties and contains many active components that might directly influence cellular processes at the gene level by increasing O-6-methylguanine-DNA methyltransferase activity that removes deleterious mutations in immune cells 14. AE prevents chemical-induced DNA-damage in animal model. Our present results suggest a likely simultaneous effect of AE treatment on multiple cellular processes in OC through modulation of multiple targets.

## Conclusion

Present study indicates for the first time an unmodified plant extract - AE treatment sensitizes highly resistant HGSOC cells and inhibits growth of these cells through modulation of key signaling molecules of angiogenesis and metastasis. AE, alone or in combination with currently used chemotherapeutic agents, may be an effective adjunct therapeutic agent for the treatment of resistant heterogenous HGSOC.

## Figures and Tables

**Table 1:** Characteristics of various HGSOC cells used.

Cell name	Histopathological sub-type	Origin	Characteristics	Resistance
TOV3041G	Human high- grade serous	Tissue	No predicted deleterious mutation identified	Isolated after Carboplatin/taxol, cisplatin/taxol treatment
OV866(2)	Human high-grade serous	Serous	TP53 mutant [-c 745 A>T (R249W)	Isolated after Carboplatin/taxol treatment
OV4453	Human high-grade serous	Serous	TP53 mutant [c375-1 G>A (splice), BRCA2 mutant [c 5857 G>T (E1953X), CSMD3 mutant (c 1937 G>C (S646T)] , RB1 mutant [c 2490-5_2490-1 del5 (splice)]	Isolated before chemotherapy treatment
OV4485	Human high-grade serous	Serous	TP53 mutant [c 818 G>A (R273H), BRCA1 mutant [c4485-1 G>T (splice).	Isolated after Carboplatin/taxol treatment

***Note:*** Heterogeneous cell lines of serous or tissue origin used for present studies were previously characterized by Fleury *et al* (2015). Specifically, OV4485 cells were isolated after carboplatin/taxol treatment while comparable OV4453 were isolated prior to chemotherapy. OV4485 carrying TP53 and BRCA1 mutations were found to be most aggressive (Fleury *et al.* 2015). Present *in vitro* studies also indicated highly aggressive and resistant nature of OV4485 cells (see Results and Discussion sections). OV4485 were selected as a representative resistant cell line for *in vivo* xenograft studies.

## Abbreviations

AE: Amla (*Emblica officinalis*) extract****
EMT: epithelial-to-mesenchymal transition
FBS: fetal bovine serum
HGSOC: high-grade serous ovarian cancers
HIF-1α: hypoxia-inducible factor 1α
IGF1R: insulin growth factor receptor 1
MTT: [3-(4,5-dimethylthiazol-2-yl)-2,5-diphenyltetrazolium bromide)]
OC: ovarian cancer
OSEM: ovarian surface epithelial Medium
